# POLICY MODIFICATION, CLINICIAN TRAINING, AND ENROLLMENT OF COMMUNITY-DWELLING OLDER ADULTS TO ADOPT A MODEL OF CARE

**DOI:** 10.1093/geroni/igz038.3277

**Published:** 2019-11-08

**Authors:** Sandra L Spoelstra

**Affiliations:** Grand Valley State University, Grand Rapids, Michigan, United States

## Abstract

Partnering with a Medicaid Home and Community Based waiver, we tests implementation strategies on adoption and sustainability of a model of care to support aging-in-place, intervening on individual capabilities and the home environment. Knowledge-to-Action model underpins the 2-arm (usual care + internal facilitation versus additive external facilitation) 3-year randomized (sites) trial statewide using an implementation strategy bundle (relationship/coalition/team building; education; interdisciplinary care/coordination; facilitation; and audit/feedback). Consolidated Framework for Implementation Research guides examining characteristics (site/RN-OT-SW/beneficiary), clinician attitude/self-efficacy, leadership/readiness to implement, and policy on adoption and sustainability; and intervention impact on beneficiary outcomes. Linear models will be used to analyze fidelity, Poisson and bootstrapping gage adoption, sustainability, fidelity, and level of facilitation. Champions (internal facilitators/supervisors; N=57) were engaged and 33.3% (n=19) completed online training (1.5 hours; 7.0 [SD 2.3] understood role; 6.8 [SD 1.8] would use training: 1-10 [lo-hi]); 100% (N=19) attended monthly coalition-building meetings. RN-OT-SWs were recruited (mail); 608 of 685 (88.8%) consented and completed online education (5.5 hours; Certified). Policymakers included quality incentives in contracts (10/1/2019) with sites for >95% certified RN-OT-SWs. Beneficiaries (N=12,000) were recruited (mail); 5% (n=608) did not participate (16.1% [n=98] poor cognition; 11.5% [n=70] nursing home/assisted living; 7.1% [n=43] no needs; 5.9% [n=36] too sick; 5.2% [n=32] many caregivers; 5.1% [n=31] inability to improve]; no English/hospitalized/other). Stages of Implementation Completion and fidelity data (September/October) will be reported. Adoption of models of care to support aging-in-place in Medicaid settings are a challenge, and this research harnesses a network (university/policymaker/supervisors/waiver sites-clinicians/beneficiaries) to improve care for vulnerable older adults.

